# The distance of the femoral neurovascular bundle from the hip joint: an intraoperative guide to reduce iatrogenic injury

**DOI:** 10.1186/s13018-018-0847-5

**Published:** 2018-06-04

**Authors:** Cyrus R. Mehta, Alex Constantinidis, Moussa Farhat, Mayuran Suthersan, Edward Graham, Andrew Kanawati

**Affiliations:** Westmead Hospital, corner of Hawkesbury and Darcy Roads, Westmead, Sydney, New South Wales 2145 Australia

**Keywords:** Total hip arthroplasty, Iatrogenic injury, Femoral neurovascular structures

## Abstract

**Background:**

Iatrogenic injury to the femoral neurovascular bundle is not uncommon during primary and revision total hip replacement (THR) and can result in permanent weakness, pain and poor function. Prevention of injury to these structures relies on a sound knowledge of their relationships to the hip joint.

**Methods:**

We studied 115 consecutive hip magnetic resonance imaging (MRI) results in order to identify objective relationships between these structures and the hip joint that can be used intraoperatively.

**Results:**

We determined that the shortest mean distances of the femoral nerve, artery and vein from the hip joint are 23.62 (standard deviation, SD = 5.44), 19.62 (SD = 4.17) and 17.47 (SD = 4.41) mm, respectively. The femoral nerve was lateral to the hip joint in 30 (55.5%) left- and 37 (60.7%) right-sided hip joints. The femoral artery was located medial to the hip joint in 28 (51.9%) left- and 34 (55.7%) right-sided hips. The femoral vein was medial to the hip joint in 52 (96.3%) left- and 58 (95.1%) right-sided hips.

**Conclusion:**

We have identified objective relationships between the hip joint and femoral neurovascular bundle that can be used with ease intraoperatively during THR. Our data show that patients with a low body weight and the elderly may be at a higher risk of iatrogenic injury due to increased proximity of the neurovascular structures to the hip. Application of this knowledge may serve to reduce the risk of iatrogenic injury to these structures and thereby improve patient satisfaction and outcomes.

## Background

Iatrogenic injury to the femoral neurovascular bundle is a rare but well documented complication of total hip replacement (THR). A recent systematic review found that up to 60% of femoral nerve injuries are iatrogenic. The incidence of injury to the femoral nerve varies in the literature between 0.2–2.4% of cases in primary THR and 1.4 to 3.8% in the revision setting [1–5]. The aetiology of injury includes direct trauma, haemorrhage/haematoma, anatomical variances, retraction and leg lengthening [6–8].

Injury to the femoral vessels has also been reported during internal fixation of intertrochanteric fractures of the femur [9]. Riouallon et al. have identified cases of iatrogenic femoral artery injury and proposed risk factors for injury that include presence of a vascular history (tobacco use, arteriopathy, bypass surgery), acetabular dysplasia, protrusion, rheumatoid arthritis, Paget’s disease and previous pelvic or acetabular fractures [10]. Injury to these structures results in poor functional outcome with the majority of patients having ongoing weakness, paraesthesia or neuropathic pain [11, 12].

Prevention of iatrogenic femoral neurovascular injury requires a sound knowledge of the anatomy of the native hip joint and constant vigilance intraoperatively to prevent inadvertent damage. There is however significant anatomic variation in the location and course of the nerve and vessels [13].

In this study, we identified objective relationships between the femoral neurovascular bundle to the hip joint based on magnetic resonance imaging (MRI) results. These relationships may prevent iatrogenic injury to the femoral neurovascular bundle adjacent to the hip joint.

## Methods

The anatomic relationships of the femoral neurovascular bundle to the hip joint during total hip replacement was studied by reviewing 115 consecutive hip and pelvis MRI results. MRIs performed at our institution within the last year were reviewed for inclusion, regardless of their indication or the patient’s underlying pathology. The majority of pelvic MRI scans performed at our institution were indicated for monitoring of patients with cancer. This explains the high incidence of cancer rather than severe arthritic hip pathology within our cohort. Only minor hip pathologies were included due to their non-deforming nature.

Exclusion criteria included patients younger than 18 years of age, a history of prior surgery to the hip and/or surrounding soft tissues, studies that revealed infiltration (neoplastic or otherwise) of the bony pelvis, severe deforming arthropathy, subluxation or dislocation of the hip joint, motion artefact and studies in which the femoral nerve and vessels were difficult to identify.

**Fig. 1:**
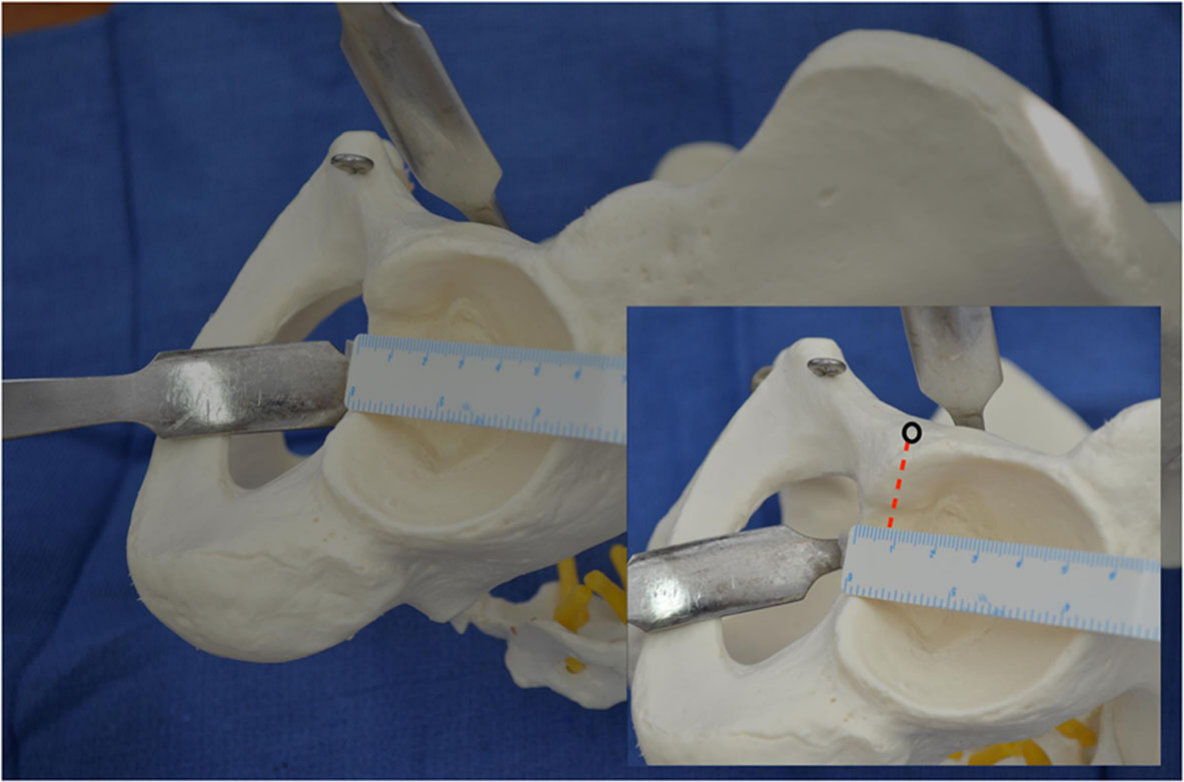
Anterior acetabular retractor in the 9 o’clock position of the left hip 8 mm superior (from the red line) to the approximate level of the acetabular teardrop (black circle)

Images were acquired using either a 3T (Siemens Magnetom Trio) or 1.5T (General Electric) MRI system. Axial proton density images were examined at the level corresponding to the widest diameter of the acetabular floor. This was routinely the axial image two slices above the teardrop on the coronal sections. This is a point that can be reproduced intraoperatively, with a slice thickness of 4 mm, making the reference point 8 mm superior to the inferomedial aspect of the acetabulum (Fig. 1).

**Fig. 2:**
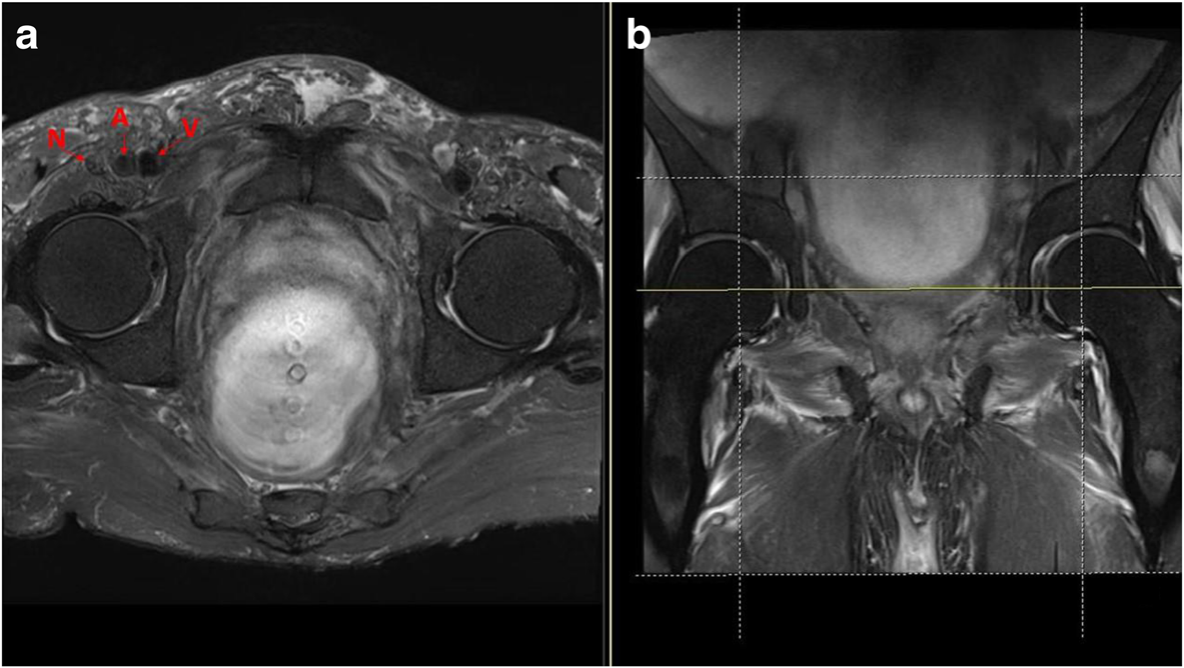
**a** Identification of structures on MRI. N femoral nerve, A femoral artery, V femoral vein. **b** Yellow line indicates the level of the axial slice used

**Fig. 3:**
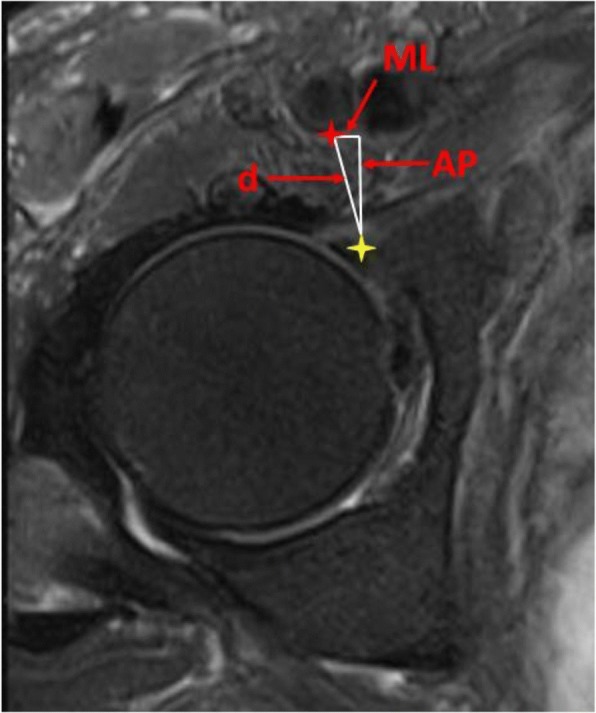
Measurement of femoral artery (red star) to the most anterior point of the hip joint at a point two slices above the teardrop (yellow star). ML mediolateral, AP anteroposterior, d shortest direct distance

The reference point defined as the hip joint, from which all measurements were made, corresponded to the anteriormost point of the anterior acetabular wall. From this point, three measurements were made with respect to each neurovascular structure in question: (femoral nerve, artery and vein) anteroposterior (AP), mediolateral (ML) and the shortest direct distance (d). The distance from the nerve and vessels was measured from their closest identifiable margins on the selected axial image (Figs. 2 and 3). The vein was identified by its medial relation, a larger lumen and thinner wall. Conversely, the artery was identified as having a smaller lumen and thicker wall. For the purpose of generating a gross three-dimensional map of the relationships, the measurements were also completed at a point 8 mm superior and 8 mm inferior to the aforementioned reference point.

Measurements were carried out and tabulated independently by three orthopaedic registrars. In the case of disagreement of more than 2 mm between measurements, the image in question was reviewed by all three registrars and a consensus reached based on a majority vote.

The mean of the measurements was used for statistical analysis. Correlations between the measurements were analysed using Pearson’s correlation coefficient (*r*). Statistical analysis was performed using SPSS version 22.0 software (IBM-SPSS, Armonk, New York). A value of *p* < 0.05 was taken as statistically significant. The study had institutional review board approval.

## Results

The study included a total of 115 hips in 64 patients with an average age of 56.8 (SD = 16.8) years. There were 61 right and 54 left hips. The average patient weight at the time of MRI was 75.5 kilogrammes (kg) (SD = 18.4 kg). The most common pathology found in the studied patients was rectal cancer (Table 1).

**Table 1:** Pathology by number of hips

Pathology	Number of hips
Normal	22
Rectal cancer	22
Vaginal/cervical cancer	17
Labral tear	11
Anal fistula/mass	8
Osteoarthritis	6
Uterine fibroid/fistula	4
Sacroiliitis	4
Gluteal pathology	3
Pelvic mass	2
Crohn’s disease	2
Spermatic cord pathology	2
Tumour of vastus lateralis	2
Ovarian cancer	2
Prostate cancer	2
Benign prostatic hyperplasia	2
Femoro-acetabular impingement	2
Bursitis	1
Hip effusion	1

**Fig. 4:**
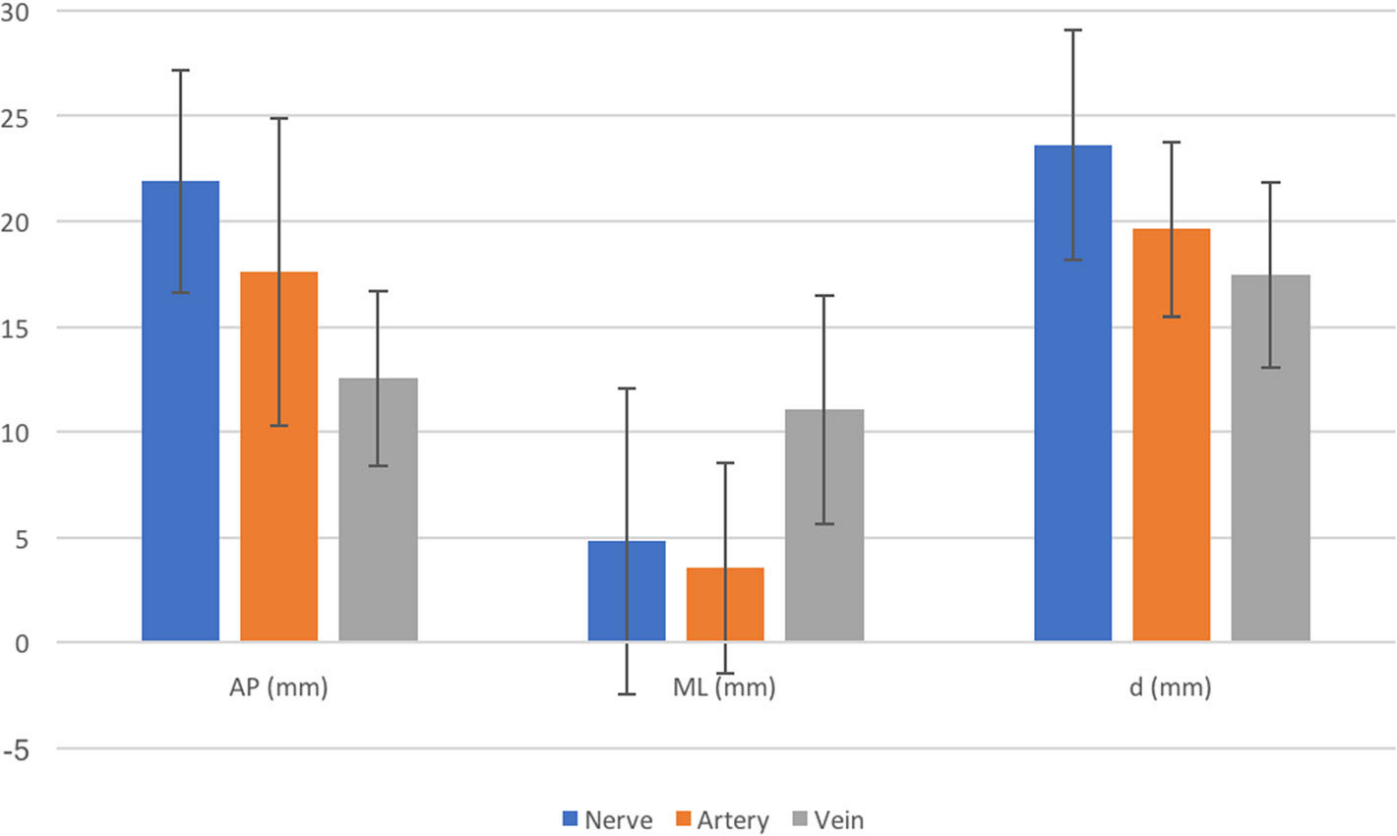
Mean distance (±standard deviation) of the femoral nerve, artery and vein from the hip joint. AP anteroposterior, ML mediolateral, d shortest direct distance

The mean AP distance of the femoral nerve from the hip joint was 21.89 (SD = 5.27) millimetres (mm). The ML distance was 4.84 mm (SD = 7.27) lateral with respect to the joint, and the shortest distance from the hip joint to the femoral nerve was 23.62 mm (SD = 5.44). It was lateral to the hip joint in 30 (55.5%) left- and 37 (60.7%) right-sided hip joints (Fig. 4).

The mean AP distance of the femoral artery was 17.61 mm (SD = 7.28), the ML distance was 3.55 mm (SD = 4.98) medial to the hip joint and the shortest distance from the artery to the hip joint was 19.62 mm (SD = 4.17). It was located medial to the hip joint in 28 (51.9%) left- and 34 (55.7%) right-sided hips. It was directly anterior (i.e. above) to the hip joint in 22 (40.7%) left- and 22 (36.1%) right-sided hips.

The mean AP distance of the femoral vein was 12.53 mm (SD = 4.17) from the hip joint and 11.05 mm (SD = 5.43) medial and the shortest distance from the artery to the hip joint was 17.47 mm (SD = 4.41). It was medial to the hip joint in 52 (96.3%) left- and 58 (95.1%) right-sided hips (Table 2).

**Table 2:** Number (and percentage) of location of the nerve, artery and vein relative to the hip joint

	Left hip			Right hip		
	*N* (%) medial	*N* (%) above	*N* (%) lateral	*N* (%) medial	*N* (%) above	*N* (%) lateral
Nerve	6 (11.1)	18 (44.4)	30 (55.5)	6 (9.8)	18 (29.5)	37 (60.7)
Artery	28 (51.9)	22 (40.7)	4 (7.4)	34 (55.7)	22 (36.1)	5 (8.2)
Vein	52 (96.3)	2 (3.7)	0 (0)	58 (95.1)	3 (4.9)	0 (0)

*N* number

The distances between the neurovascular bundle and the hip were also measured at a point 8 mm superior and 8 mm inferior (i.e. at the level of the teardrop) to our described reference point. The mean (±SD) differences in distance for the measured relationships both 8 mm superior and 8 mm inferior to our reference point are given in (Table 3).

**Table 3:** Distances between neurovascular bundle 8 mm superior and inferior to the described reference point (mm ± SD)

	V (AP)	V (ML)	V (d)	A (AP)	A (ML)	A (d)	N (AP)	N (ML)	N (d)
Above	0.75 (1.5)	2 (1.83)	2.25 (0.96)	1.75 (0.96)	0.5 (0.58)	3.25 (1.71)	1.25 (1.89)	4 (3.56)	4 (3.27)
Below	1.75 (0.5)	2.75 (0.96)	1.25 (0.5)	0.75 (0.5)	1 (1.4)	0.75 (0.5)	2.75 (3.59)	2.25 (2.87)	2.5 (3.3)

V vein, A artery, N nerve, AP anteroposterior, ML mediolateral, d shortest distance

There is a statistically significant correlation between patient weight and the shortest distance of the artery and nerve to the hip joint. The strength of correlation is weak-moderately positive for both the distance of the artery (*r* = 0.388, *p* < 0.001, df = 67) and distance of the nerve (*r* = 0.445, *p* < 0.0001, df = 67).

There are very strong, statistically significant negative correlations between the age of the subject and the shortest distances for the vein (*r* = − 0.371, *p* < 0.001, df = 114) and artery (*r* = − 0.380, *p* < 0.001, df = 114). Similarly, there is a weak negative correlation that is strongly statistically significant between the age of the subject and the shortest distance between the nerve and the hip joint (*r* = − 0.253, *p* = 0.006, df = 114).

**Fig. 5:**
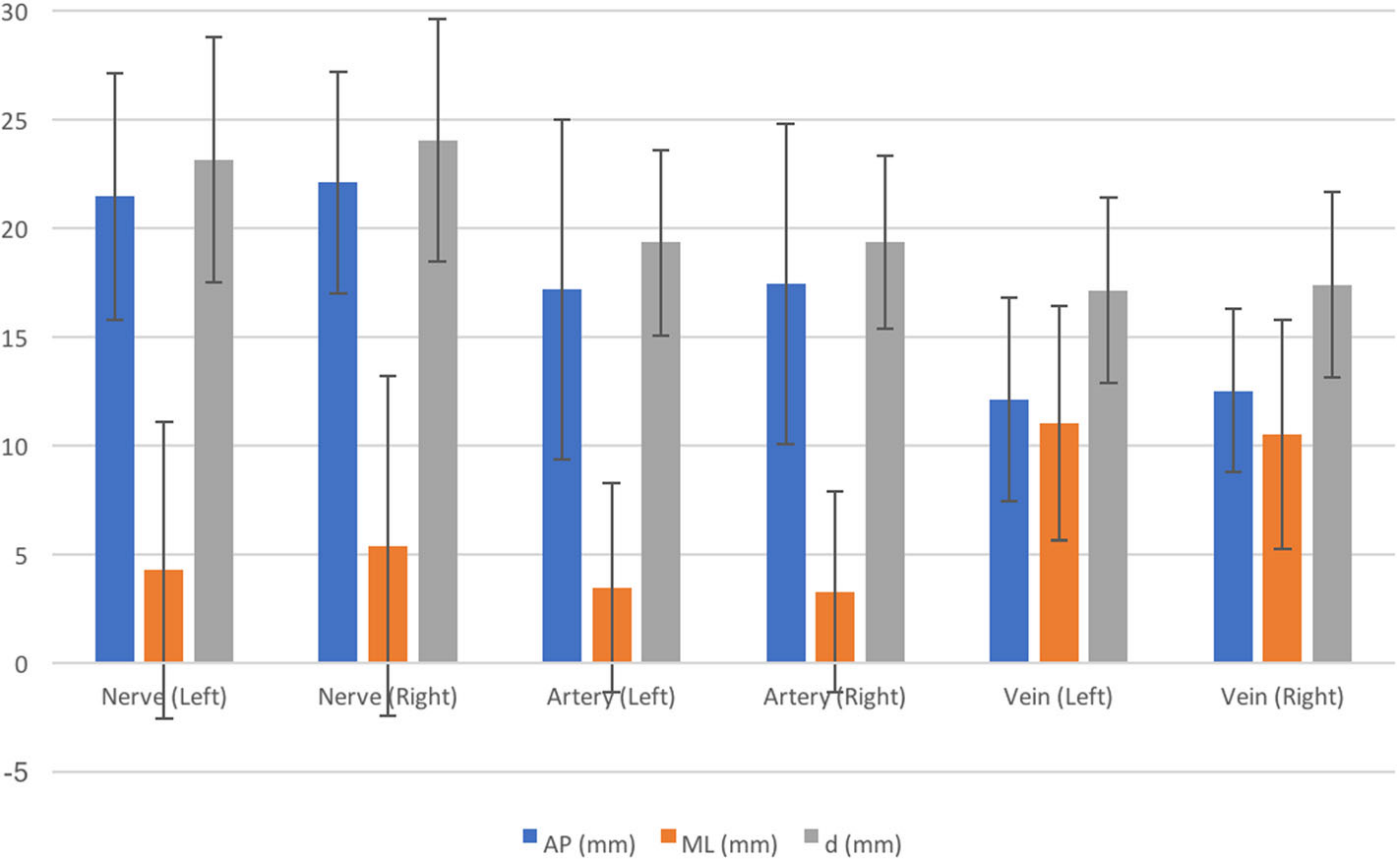
Comparative distances between left and right femoral neurovascular structures from the hip joint. AP anteroposterior, ML mediolateral, d shortest direct distance

There was no statistically significant difference between left- and right-sided measurements (correlation coefficient > 0.7, *p* < 0.05) (Fig. 5).

## Discussion

Our study has examined a large sample size of patients in order to provide objective, anatomic relationships that can be used with ease intraoperatively in order to avoid iatrogenic neurovascular injury during THR.

In this study, we assessed the AP, ML and shortest distances of the femoral nerve, artery and vein from the hip joint on 115 proton density MRI studies. We determined that the shortest mean distances of the femoral nerve, artery and vein from the hip joint are 23.62 (SD = 5.44), 19.62 (SD = 4.17) and 17.47 (SD = 4.41) mm, respectively. There was no statistically significant difference in the AP, ML or shortest distance measurements when comparing the left- and right-sided hips. Shubert et al. have previously described the relationship of the sciatic nerve to retractors during THR in 48 patients and found that women are at a higher risk of suffering iatrogenic neurovascular injury [14]. Similarly, Wang et al. identified distances between bony landmarks and the femoral nerve in hip MRIs and identified that shorter patients will be at higher risk for neural injury intraoperatively [15]. Our study design involved the retrospective analysis of MRI images and patient data that were collected for purposes other than completing our study. In our institution, it is not routine to record patient height prior to conducting an MRI and therefore this information was unavailable to us for inclusion in our analysis. Hence, we cannot comment on any relationship that may or may not exist between height and the distance of the femoral neurovascular structures to the hip joint. This should be a focus of future research. The femoral nerve was found to lie lateral to the hip in 55–60% of cases, the femoral artery was medial to the hip in 51–56% of cases and the femoral vein was medial to the hip in 95–96% of cases. These findings support the previously described relationships of the neurovascular structures anterior to the hip joint [16]. The reference point used in our study is located 8 mm superior to the acetabular teardrop. During acetabular exposure, three to four retractors are placed around the acetabulum in order to increase visualization. The anterior retractor is described as being passed over the anterior rim of the acetabulum [17]. There are three commonly described anterior acetabular retractor positions during total hip replacement. The anterior wall retractor is placed at the 3 o’clock position for the right hip or 9 o’clock for the left. This position was estimated to lie at a point 8 mm proximal to the acetabular teardrop as illustrated in Fig. 1.

Using the above relationships allows us to establish safe zones that may be used intraoperatively to prevent iatrogenic injury. For example, based on our findings that the femoral nerve has a mean ML distance of 4.81 (SD = 7.27) mm lateral to the hip joint, it can be seen that pericapsular local anaesthetic injection should be done within 11 mm of the hip joint in order to prevent inadvertent injection into the femoral nerve. Similarly, when performing a capsular release, if one uses the mean AP distance of the nerve from the hip joint, it can be seen that this release should not be performed to a depth greater than 27 mm from the aforementioned reference point. Using this method, safe zones for the artery and nerve can also be calculated. All of the measurements both above and below the reference point were found to fall within the standard deviations of our previously described relationships. It can therefore be seen that even if the reference point for measurement is altered, these relationships are still valid. In spite of this, for the reasons outline above, the reference point chosen in this study is the most applicable and pragmatic for use intraoperatively.

Making measurements above and below the reference point gives rough three-dimensional description of the neurovascular bundle’s relationship to the multiple reference points. However, the relationships are similar at all three levels, so the need for a three-dimensional description is less. More sophisticated software could be used in future studies to confirm this relationship.

There was a statistically significant positive correlation between the weight of the patient and the shortest distance of the femoral artery and nerve to the hip joint. Similarly, there was a negative correlation between age of the subject and the shortest distances for the vein and artery. That is, the neurovascular structures were found to be closer to the hip joint in elderly individuals and those with lower body weight; thus, these patients may be at a higher risk of iatrogenic injury during THR [18].

Clinical applications of these results include when performing anterior capsular releases during the posterior approach to the hip. Additionally, with the increasing use of periarticular local anaesthetic infiltration during total hip replacement, these relationships can be helpful to undertake safe injection, especially around the femoral nerve as aspiration of blood from the artery or vein is not possible when injecting close to the nerve [19].

Given the study population, the findings of this study are only relevant in cases where the anatomy is not distorted. The majority of patients included in the study did not have hip pathology and are not necessarily reflective of patients undergoing THR. They are not relevant in situations of revision arthroplasty, severe deformity arthropathy, dysplasia with subluxation or dislocation or fracture of the femoral neck. In the arthroplasty situation, after femoral neck osteotomy, the above measurements will be distorted. However, once anatomy is restored with prosthetic implantation, assuming anatomic alignment of the prosthesis is re-established after implantation, one can use the described relationships again. The distances described above were measured from MRIs where the subjects were laying supine; it is unknown how these distances will be affected (if at all) once the patients are in a lateral decubitus position for THR. Lastly, the described distances are based on MRI findings and may vary during an open surgical approach to the hip joint. Further research is necessary to determine how the anatomy of the femoral neurovascular bundle changes in relation to the acetabulum and femur when the limb is positioned for acetabular and femoral preparation.

## Conclusion

We have identified objective relationships between the hip joint and femoral neurovascular bundle that can be used with ease intraoperatively during THR. Our data show that patients with a low body weight and the elderly have a greater proximity of the neurovascular structures to the hip joint and therefore may be at a higher risk of iatrogenic injury. Application of this knowledge will serve to reduce the risk of iatrogenic injury to these structures and thereby improve patient satisfaction and outcomes.

## Abbreviations

AP: Anteroposterior
d: Distance
ML: Mediolateral
MRI: Magnetic resonance imaging
*p*: *p* value
*r*: Pearson’s correlation coefficient
SD: Standard deviation
THR: Total hip replacement

## References

[1] Kretschmer T, Heinen CW, Antoniadis G, Richter H, König RW (2009). Iatrogenic nerve injuries. Neurosurg Clin N Am.

[2] Farrell CM, Springer BD, Haidukewych GJ, Morrey BF (2005). Motor nerve palsy following primary total hip arthroplasty. J Bone Joint Surg.

[3] Weale AE, Newman P, Ferguson IT, Bannister GC (1996). Nerve injury after posterior and direct lateral approaches for hip replacement. A clinical and electrophysiological study J Bone Joint Surg.

[4] Navarro RA, Schmalzried TP, Amstutz HC, Dorey FJ (1995). Surgical approach and nerve palsy in total hip arthroplasty. J Arthroplast.

[5] Simmons C Jr, Izant TH, Rothman RH, et al. Femoral neuropathy following total hip arthroplasty. Anatomic study, case reports, and literature review. J Arthroplasty. 1991;6(6 Suppl):S57–66. 1663537

[6] Schmalzried TP, Amstutz HC, Dorey FJ (1991). Nerve palsy associated with total hip replacement. Risk factors and prognosis. J Bone Joint Surg.

[7] McConaghle FA, Payne AP, Kinninmonth AWG (2014). The role of retraction in direct nerve injury in total hip replacement. Bone Joint Res.

[8] Fox AJ, Bedi A, Wanivenhaus F, Sculco TP (2012). Femoral neuropathy following total hip arthroplasty review and management guidelines. Acta Orthop Belg.

[9] Segal D, Yaacobi E, Marom N, Feldman V, Aliev E, Palmanovich E, Brin YS (2017). The incidence of life threatening iatrogenic vessel injury following closed or open reduction and internal fixation of intertrochanteric femoral factures. Int Orthop.

[10] Riouallon G, Zilber S, Allain J (2009). Common femoral artery intimal injury following total hip replacement. A case report and literature review. Orthopaedics & Traumatology: Surgery & Research.

[11] Johanson NA, Pellicci PM, Tsairis P, Salvati EA (1983). Nerve injury in total hip arthroplasty. Clin Orthop Relat Res.

[12] Schmalzried TP, Amstutz HC, Dorey FJ (1991). Nerve palsy associated with total hip replacement: risk factors and prognosis. J Bone Joint Surg [Am].

[13] Ward R (2016). Hip joint. Bergmans comprehensive encyclopedia of human anatomic variation.

[14] Shubert D, Madoff S, Milillo R, Nandi S (2015). Neurovascular structure proximity to acetabular retractors in total hip arthroplasty. J Arthroplast.

[15] Wang T, Chen H, Tsai C, Hsu H, Lin T. Distances between bony landmarks and adjacent nerves: anatomical factors that may influence retractor placement in total hip replacement surgery. J Orthop Surg Res. 2016;11(1) 10.1186/s13018-016-0365-2. 10.1186/s13018-016-0365-2 PMC4794908 26984637

[16] Neurovascular Anatomy in Interventional Neuroradiology. (2015). Neurovascular Anatomy in Interventional Neuroradiology. 10.1055/b-0035-129450.

[17] Hoppenfeld S, Boer PD, Buckley R (2017). Surgical exposures in orthopaedics: the anatomic approach.

[18] Yacub J, Bradford Rice J, Dillingham T (2009). Nerve injury in patients following hip and knee arthroplasties and knee arthroscopy. Am J Phys Med Rehabil.

[19] Starks I, Wainwright T, Middleton R. Local anaesthetic infiltration in joint replacement surgery: what is its role in enhanced recovery? International Scholarly Research Notices Anaesthesiology. 2011; 10.5402/2011/742927.

